# Unveiling the Hidden: A Case Report on Altemeier’s Surgical Triumph in Rectal Prolapse With Involvement of the Small Bowel

**DOI:** 10.7759/cureus.81206

**Published:** 2025-03-25

**Authors:** Kavita Jadhav, Ami Gandhi, Rukmini Waghmare, Dhanashri Sidam, Shubham More

**Affiliations:** 1 General Surgery, Grant Government Medical College and Sir J.J. Group of Hospitals, Mumbai, IND

**Keywords:** altemeier's procedure, general surgery, perineal approach, procidentia, rectal prolapse, small intestine, surgical case reports

## Abstract

Full-thickness rectal prolapse is a condition where the rectum or rectosigmoid protrudes through the anus. This condition can severely impact quality of life, requiring accurate diagnosis and treatment. Small bowel involvement is rare. This case report presents a 26-year-old male patient with complete rectal prolapse that could not be reduced through conservative management. Due to the irreducibility of the prolapse, even under spinal anesthesia, Altemeier's repair was ultimately performed. During surgery, loops of the small intestine were found along with the prolapsed segment of the rectum and sigmoid colon. Surgical management of rectal prolapse includes abdominal approaches such as laparoscopic or open ventral mesh rectopexy for a carefully selected subset of younger patients and perineal approaches such as Delorme's and Alteimeier's for elderly patients. In this case, despite the patient’s young age, Altemeier's procedure was chosen due to the irreducibility of the prolapse. This case highlights the necessity of tailored surgical approaches for rectal prolapse. Individualized patient care and thorough evaluation of surgical options are crucial in managing this condition, ensuring the best possible patient outcomes.

## Introduction

Rectal prolapse is defined as the concentric protrusion of full or partial thickness of the rectum or rectosigmoid via the anus. This condition is estimated to occur in less than 0.5% of the population, with a higher frequency observed in elderly females [1]. Complete prolapse involves the entire rectal wall protruding outside the anus, characterized by concentric folds [2]. Incomplete prolapse, also known as occult rectal prolapse or internal rectal intussusception, refers to the protrusion of the rectal wall confined within the anal canal [3]. It is essential to distinguish mucosal prolapse from a full-thickness rectal prolapse, as their surgical treatments vary. Rectal prolapse significantly affects the quality of life of individuals [4].

Rectal prolapse is often associated with anatomical abnormalities such as weakness of the levator ani muscles, a redundant sigmoid colon, and weakened rectal attachments. Surgical intervention is indicated when conservative management fails, aiming to restore normal physiology, improve continence, and relieve constipation. Various surgical options exist, including perineal and abdominal approaches such as proctosigmoidectomy and rectopexy, but the choice of procedure depends on patient comorbidities, age, and bowel function.

Herein, we present a case of a 26-year-old male with rectal prolapse, managed surgically with Altemier’s repair after failed conservative measures. This paper reviews the clinical presentation, diagnostic challenges, and therapeutic strategies associated with rectal prolapse, along with the involvement of the sigmoid colon and small bowel, emphasizing the importance of tailored surgical interventions in achieving favorable outcomes.

## Case presentation

A 26-year-old male presented to the emergency department with the primary complaint of a mass protruding from the rectum, which he had been unable to reduce manually for six hours before admission. The patient reported a history of similar symptoms since the age of 12, which he had previously managed by manually reducing the prolapse and by conservative management. Other than the presenting symptoms, the patient had no other significant medical or surgical history and had no comorbidities. Upon admission, he also experienced non-bilious vomiting and dull, aching per rectal pain.

**Figure 1:**
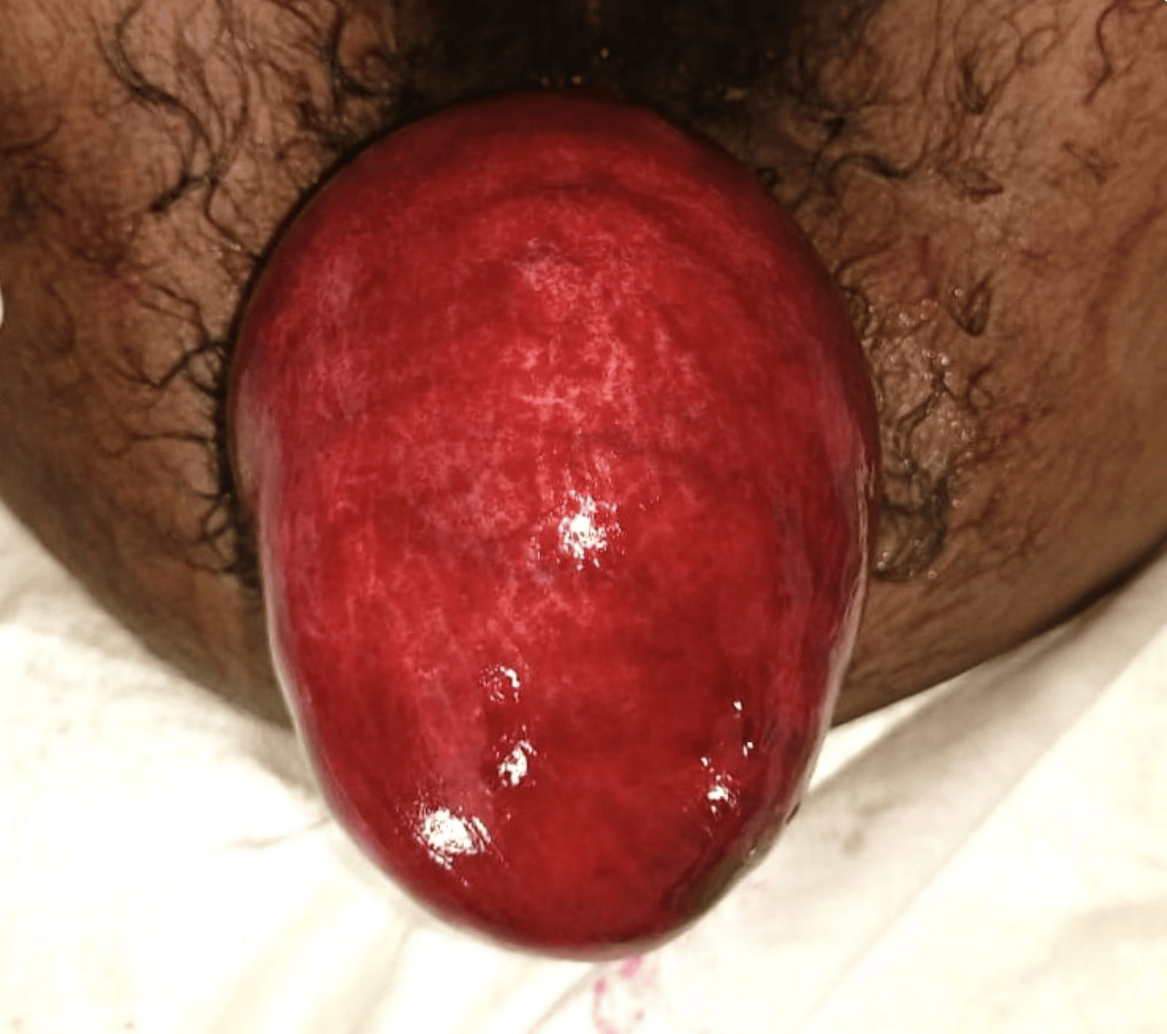
Per rectal examination showing full-thickness rectal prolapse with congested and edematous rectum

Physical examination revealed tachycardia, with an otherwise normal abdominal examination. On inspection and anorectal examination, a 15 cm protrusion of edematous, congested, and dusky-colored concentric rings of rectal mucosa was observed, confirming a full-thickness rectal prolapse (Figure 1). Radiographic imaging of an erect abdomen and chest showed no significant findings. Despite multiple attempts using the Trendelenburg position, glycerin, and manual reduction, the prolapsed rectal mucosa could not be reduced after several hours. Given concerns for possible strangulation, a decision was made to proceed with surgical intervention. Spinal anesthesia was administered, but subsequent manual reduction attempts remained unsuccessful.

**Figure 2:**
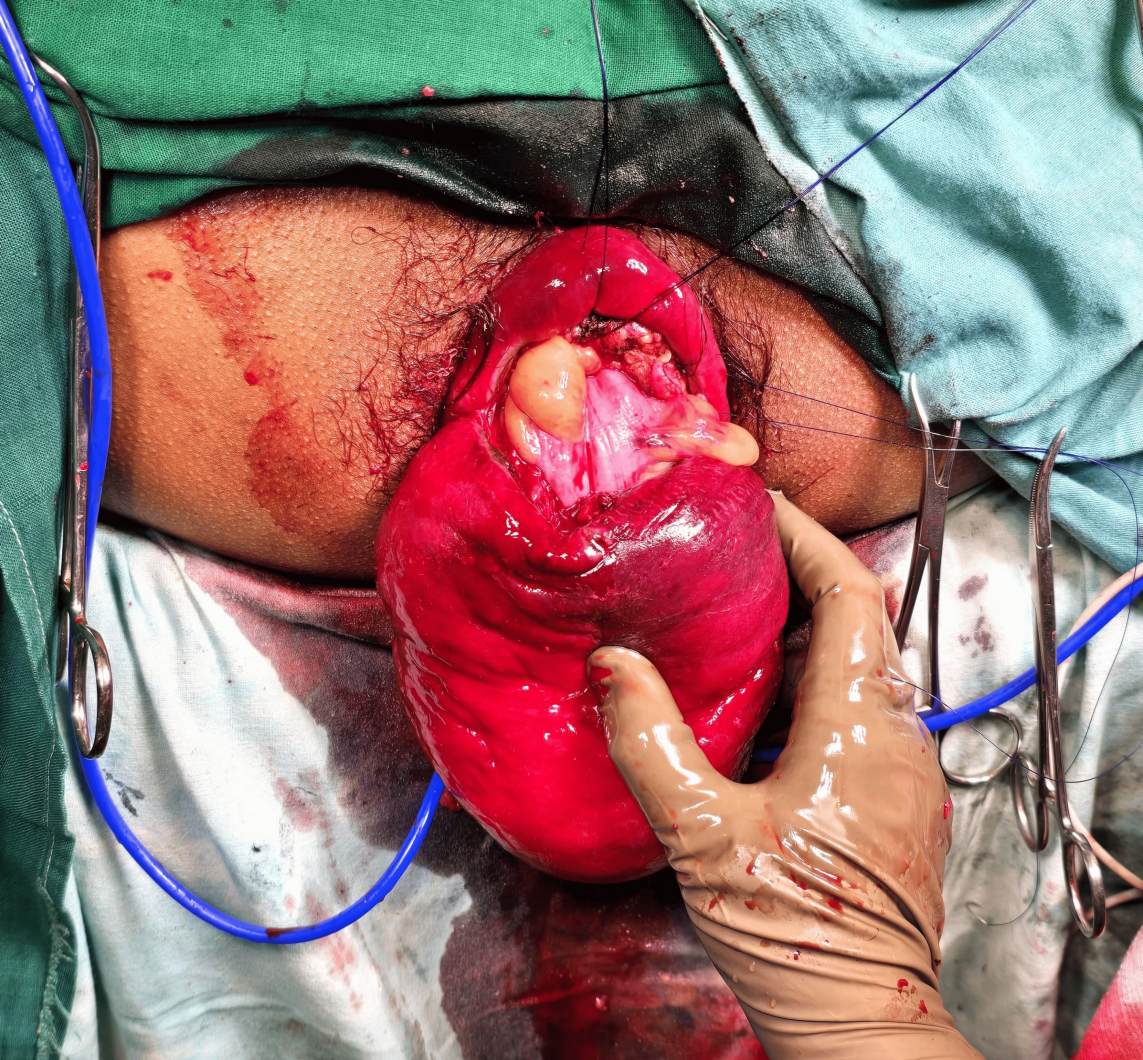
Intraoperative Altemier’s procedure initially depicting the sigmoid colon as the content

**Figure 3:**
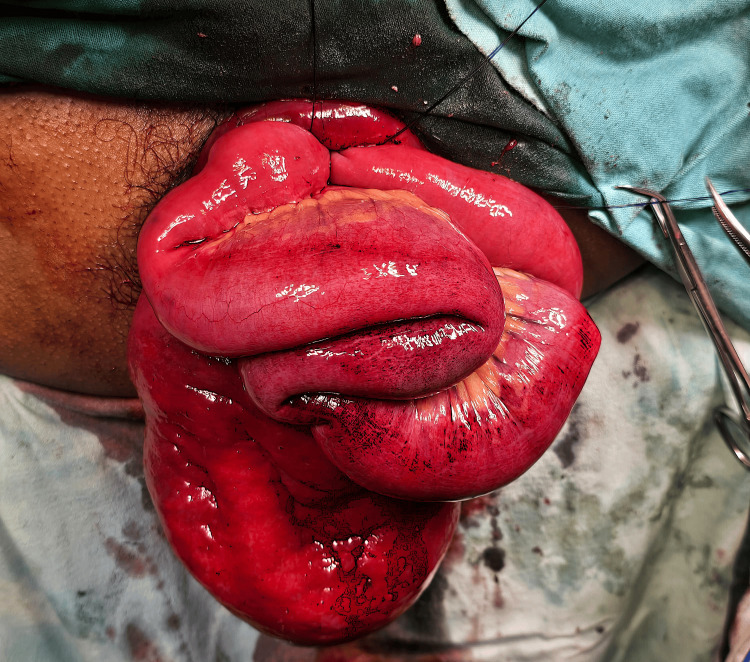
Loops of small intestine seen as content of the prolapsed rectal sac

**Figure 4:**
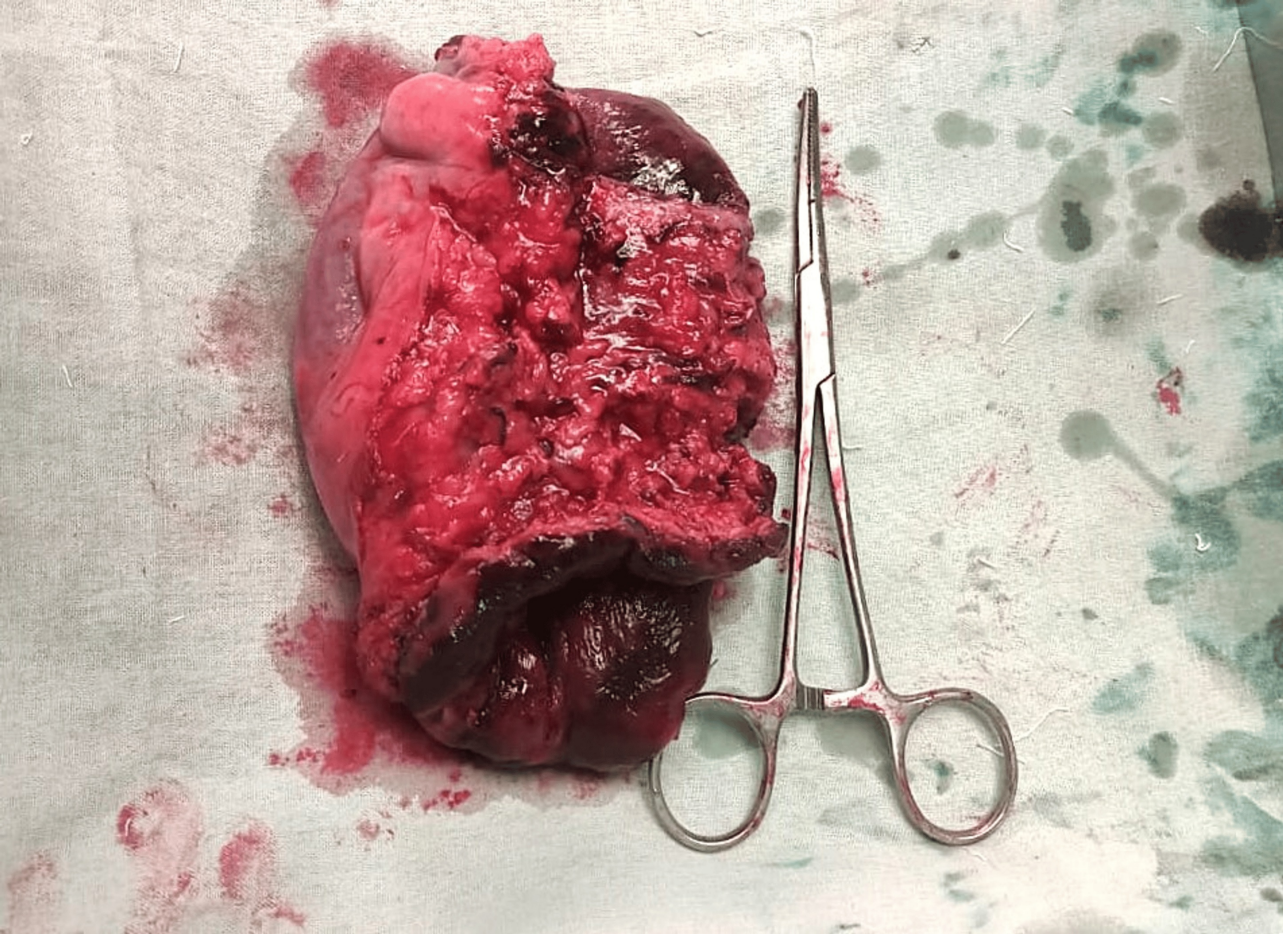
The resected specimen of sigmoid colon and proximal rectum

**Figure 5:**
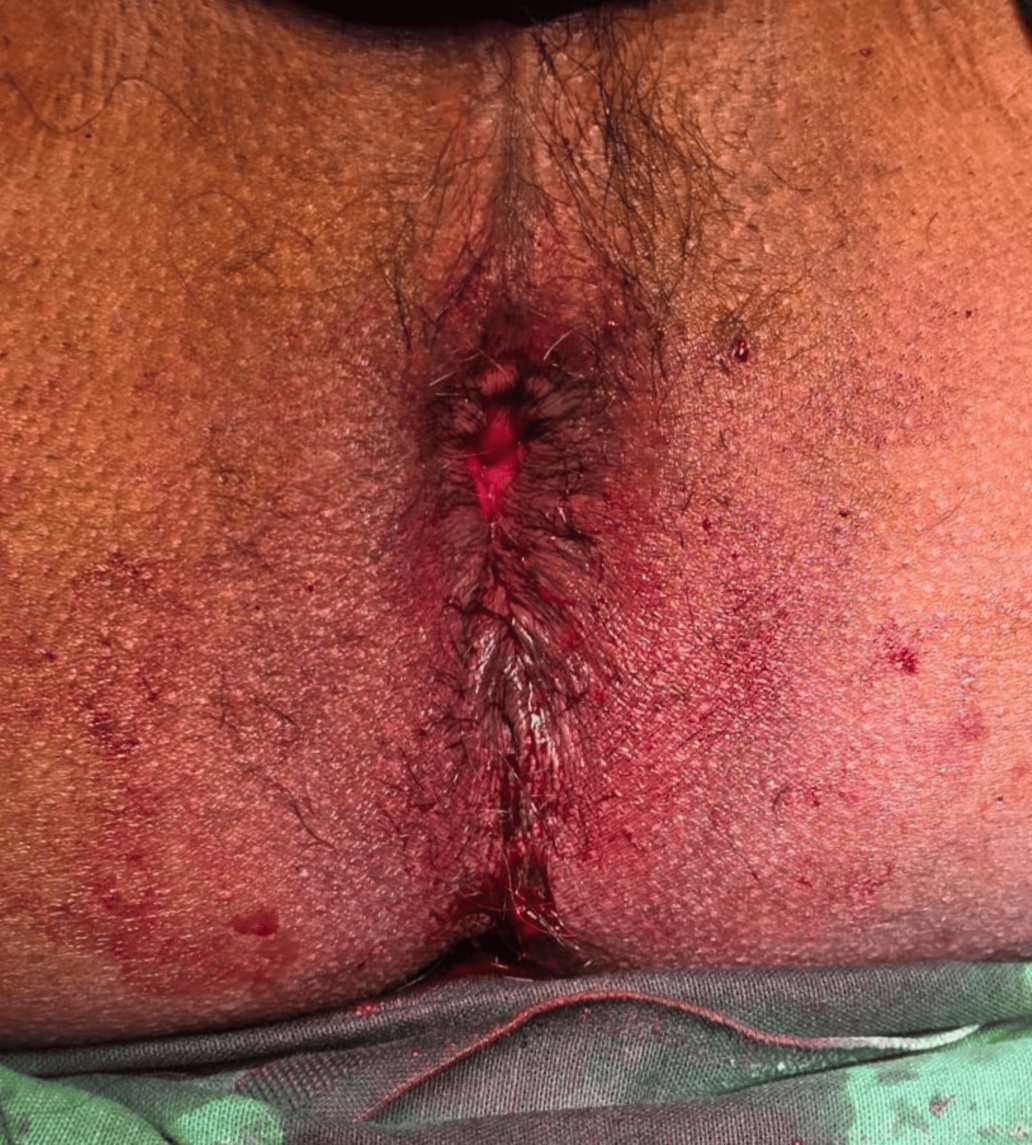
Postoperative image depicting resolution of rectal prolapse after Altemeier’s procedure

Written and informed consent was taken prior to the surgery. The patient underwent Altemier’s repair in which a circumferential incision was made on the prolapsed rectal mucosa 2 cm above the dentate line using an ultrasonic scalpel (Figure 2). Initial exploration revealed the presence of the sigmoid colon with mesorectum. However, further dissection uncovered loops of the small intestine within the prolapsed area, which were unremarkable (Figure 3). The small bowel loops appeared normal in shape and color. After reduction of the small bowel, the mesorectum and mesocolon were ligated. A 12 cm proctosigmoidectomy was performed (Figure 4), followed by coloanal anastomosis using 3-0 polydioxanone simple interrupted absorbable sutures. Anal tone was assessed and found to be patulous, prompting the placement of a Thiersch stitch to improve anal tone and prevent incontinence (Figure 5). Histopathological analysis of the resected specimen revealed chronic inflammation. The patient tolerated the procedure well, with no intraoperative or immediate postoperative complications. A gradual diet was introduced, and patient was advised pelvic floor exercises. The patient was discharged on postoperative day 7. Follow-up on postoperative days 14, 21, and 60 showed no difficulties in defecation or recurrence.

## Discussion

Rectal prolapse is associated with various anatomical abnormalities, such as separation of the levator ani muscles, an unusually deep cul-de-sac, a redundant sigmoid colon, a lax anal sphincter, and weakened or lost rectosacral attachments [5]. A comprehensive evaluation is essential during the initial assessment, focusing on potential symptoms of constipation and fecal incontinence. It is also crucial to assess for symptoms indicative of anterior compartment prolapse, including urinary incontinence and vaginal or uterine prolapse.

Surgical treatment aims to restore normal rectal anatomy and function by correcting the prolapse, improving continence, and alleviating constipation. In cases where both genital and rectal prolapse coexist, a multidisciplinary surgical approach is often required. Surgery should be considered in patients who have not responded to conservative measures and when symptom relief is expected [6].

Surgery remains the primary treatment for rectal prolapse [7]. Available surgical options include anal encirclement, mucosal resection, perineal proctosigmoidectomy, and rectopexy, with or without anterior resection. Procedures involving synthetic or biological meshes, such as ventral rectopexy, are commonly recommended [8]. Laparoscopic approaches have also been explored, particularly for ventral rectopexy with mesh in elective surgeries [9]. The choice of surgical approach is based on patient comorbidities, age, bowel function and surgeon expertise [9].

Reports of prolapsed rectum with concurrent sigmoid colon and small bowel involvement are rare in the literature. Cases reported by Kumar et al. [10], Wrobleski et al. [11], Berwin et al. [12], and Sahib et al. [13] described spontaneous rupture of the prolapsed rectum, resulting in small bowel evisceration through the anus due to elevated intra-abdominal pressure. Most of these cases involved rupture at the rectosigmoid junction, leading to small bowel evisceration, and were treated with exploratory laparotomies. However, Berwin et al. [12] and Sahib et al. [13] used a perineal approach for elderly patients who were unfit for major surgery.

In the present case, the prolapsed rectum was irreducible, and despite attempts at manual reduction under anesthesia, the decision was made to proceed with surgery using a perineal approach. Altemeier’s procedure was performed, during which small bowel loops and the sigmoid colon were found within the prolapsed rectum. The small bowel loops were successfully reduced, and coloanal anastomosis was performed perianally.

This is the first reported case of an irreducible rectal prolapse in a 26-year-old patient, without obvious rupture of the rectosigmoid junction or rectal tear, but with small bowel loops and the sigmoid colon. The case was successfully managed with the perineal Altemeier’s procedure.

## Conclusions

Understanding the distinctions between various surgical methods to manage rectal prolapse is crucial for selecting the most appropriate treatment strategy tailored to each patient's specific condition and overall health status. Our case report highlights the significance of personalized care and emphasizes the importance of a comprehensive evaluation of all available surgical options to achieve optimal outcomes in the management of rectal prolapse.
